# The *In Vitro* Effect of Ivermectin on the Activity of Trehalose Synthesis Pathway Enzymes and Their mRNA Expression in the Muscle of Adult Female *Ascaris suum* (Nematoda)

**DOI:** 10.1155/2014/936560

**Published:** 2014-10-27

**Authors:** Małgorzata Dmitryjuk, Elżbieta Łopieńska-Biernat, Ewa Anna Zaobidna

**Affiliations:** Biochemistry Department, Faculty of Biology and Biotechnology, University of Warmia and Mazury, Oczapowski 1A Street, 10-719 Olsztyn, Poland

## Abstract

The *in vitro* effect of ivermectin lethal dose on the activity of trehalose-6-phosphate synthase (TPS) and phosphatase (TPP) and the expression of their mRNA (*tps1, tps2*, and *tpp* genes) in the muscle of adult female *Ascaris suum* was investigated. The presence of ivermectin in the medium caused a decrease in TPS and TPP activities during the experiment compared with the start and control groups. The exception was the group of worms grown for 8 hours in a IVM solution, in which there was a little higher TPS activity than in the control. Real-time qPCR analysis showed reduced expression of *tps1* and *tps2*, and unchanged *tpp* expression after 20 hours of incubation relative to the expression at time zero. Relative to the appropriate control groups, the expression of *tps2* gene was slight increased but the other two genes were reduced after 8-hours of IVM-treatment. Then the expression of all three genes was lower at the end of cultivation. The level of gene expression was positively correlated with the activity of specific enzymes. In the case of *tpp* gene there was only a weak correlation. Prolonged exposure to ivermectin was effective in lowering TPS and TPP activity and their mRNA expression. However, the drug did not block the pathway.

## 1. Introduction

Ivermectin (IVM) is the 22,23-dihydroderivative of avermectin B1, a strong macrocyclic lactone produced by an actinomycete,* Streptomyces avermitilis* [1]. The action of this drug appears to depend on its effect on nerve impulse conduction [2]. Although previously it was thought that IVM acts via *γ*-aminobutyric acidergic (GABAergic) regulation [1], it has been shown that IVM induces an influx of chloride ions through channels that are not regulated by the neurotransmitter GABA. There is ample evidence that GABA plays a little part in the antiparasitic action of IVM; for example, GABA-linked Cl^−^ channels in cell membranes of* Ascaris suum* are less sensitive to IVM than are other types of channel [3]; specific IVM-binding proteins in cell membranes of* Caenorhabditis elegans* and* Drosophila melanogaster* have been identified [4]; and also IVM is insensitive to GABA but sensitive to the neurotransmitter l-glutamate in specially prepared oocytes of the frog* Xenopus laevis* after* C. elegans* mRNA injection [5, 6]. It may be possible that IVM acts on a novel type of ion channel [2].

Although the mechanism of IVM binding is unclear, it is known that it is collected in the outer monolayer of the muscle membrane and following an irreversible change in membrane potential the muscles of parasites are paralysed [7]. IVM is absorbed into the blood of the host after oral administration and is almost completely excreted in the faeces. Maximum plasma concentrations are reached approximately 4 hours after oral administration. The half-life of IVM is about 12 hours, but trace amounts of the drug are present in the blood of the host up to 3 days after administration [2]. Due to the broad spectrum of activity and large safety margin, IVM has become a medicine used for the control of nematodes and arthropod parasites in cattle, horses, sheep, goats, dogs, pigs, and humans [1, 2, 8].

The effect of IVM and other nonbenzimidazole compounds on embryonation and infectivity of* A. suum* eggs has been studied. These studies have shown that the anthelmintic treatment of pigs with IVM has only a limited effect on both embryonation and infectivity of* A. suum* eggs isolated from expelled worms [9]. On the other hand, the efficacy of the drug in inactivation of larval and adult form of* A. suum* is high, nearing 100% [10, 11].

In the present study we decided to investigate the* in vitro* influence of IVM on trehalose synthesis in muscles of adult female* A. suum*. Trehalose (*α*-d-glucopyranosyl-(1,1)-*α*-d-glucopyranoside) in nematodes is usually present at a higher concentration than the free glucose [12, 13] and has a number of important functions: it protects cellular structures during periods of stress such as high osmotic pressure, drying, and freezing; it provides energy and is the major circulating sugar; it is also important in the process of egg hatching [14–16]. In most eukaryotes, including nematodes, trehalose synthesis is catalysed by the action of two enzymes: trehalose-6-phosphate (T6P) synthase (TPS;* EC* 2.4.1.15), which catalyses the transfer of glucose from uridine diphosphate glucose (UDPG) to glucose-6-phosphate (G6P) to produce T6P, and T6P phosphatase (TPP;* EC* 3.1.3.12), which converts T6P to free trehalose and inorganic phosphate [15]. The activity of both enzymes participating in trehalose synthesis has been confirmed in the muscles, haemolymph, and reproductive system of* A. suum* [13]. TPS was isolated from muscles of* A. suum* and its properties have been detected. Also two genes encoding TPS,* tps1* (JF412033.2) and* tps2* (JF412034.2), were isolated and sequenced from muscles of the parasite [17]. The activity and preliminary properties of TPP (optimum of pH and temperature, thermostability, activators, and inhibitors) were detected in muscles of* A. suum* [18]. In the nematodes, TPP was first identified in* C. elegans* in a forward genetic screen for intestinal defects where the loss of* tpp* (*gob-1*) function resulted in early larval lethality due to a blocked intestinal lumen and consequent starvation [19]. Kushwaha et al. [20] reported on the cloning, expression, and purification of* Brugia malayi* TPP that was found to be an unusual phosphatase. These researchers have shown that* tpp* gene silencing was lethal for stage 3 (L_3_) larvae of* B. malayi* and that those defined lethal effects resulted in impaired* in vivo* development [21]. The World Health Organization has included the* B. malayi* TPP enzyme in the priority list of prospective antifilarial drug targets for lymphatic filariasis [22]. Therefore, the trehalose synthesis pathway in* A. suum* may play an important role in therapeutic aims and this pathway can be a target for antiascariasis drugs, especially since the pathway does not occur in mammalian hosts of* Ascaris* sp. In the current study, we evaluated whether IVM, a well-known antiparasitic drug, affected the activity of trehalose synthesis enzymes and expression of their mRNA. We try to find answers to the following questions: is roundworms trehalose metabolism activated to protect against lethal effects of the drug? At what level is this done? Is there a positive correlation between the activity of the trehalose synthesis pathway enzymes and their mRNA expression?

## 2. Materials and Methods

### 2.1. Chemicals

Purified bovine serum albumin (BSA), T6P dipotassium salt, uridine 5′-diphosphoglucose disodium salt from* Saccharomyces cerevisiae* (UDPG), d-glucose-6-phosphate disodium salt hydrate (G6P), and alkaline phosphatase from bovine intestinal mucosa (lyophilized powder, 10–30 defined enzyme activity (DEA) units/mL solid), penicillin G sodium salt, nystatin, IVM, 4-(2-hydroxyethyl)-piperazine-1-ethanesulfonic acid (HEPES), agarose, and ethidium bromide were purchased from Sigma-Aldrich (Germany and USA). Total RNA Kit, TranScriba Kit, and SYBR Green B PCR-MIX Taq were obtained from A&A Biotechnology (Poland). The components of 0.1 M acetic acid-ammonia buffer and 0.1 M phosphoric buffer (resp., pH 4.2 and pH 7.0) (acetic acid, ammonia, NaH_2_PO_4_, and Na_2_HPO_4_  × 7H_2_O_2_) and of* Ascaris* Ringer's Solution (ARS) medium (KCl, CaCl_2_  × 2H_2_O, MgCl_2_  × 6H_2_O, NaCl, and sodium acetate) were of high purity grade and were purchased from Chempur (Poland) and P.P.H. Stanlab (Poland).

The water used for the analysis was deionized with the use of a Direct-Q Ultrapure UV3 Water System (EMD Millipore, USA). The water, media, sodium saline, and surgical instruments were sterilized using a Classic Standard Prestige Medical autoclave (Ma-Je-R, Poland).

### 2.2. Acquisition of Material and* In Vitro* Cultures

Living mature female roundworms (*Ascaris suum* Goeze, 1782) were isolated from the intestines of pigs in a nearby slaughterhouse and transported in ARS medium [22] within 1 hour of collection. ARS medium was formulated from 25 mM potassium chloride, 15 mM calcium chloride, 10 mM magnesium chloride, 4 mM sodium chloride, and 125 mM sodium acetate and next buffered to pH 7.4 using 5 mM HEPES. In the laboratory, pig roundworms were washed in sterile saline and next in ARS medium containing antibiotics (1.2 million units of penicillin and 1 g of nystatin per litre) for 4 hours. This was followed by two equal* in vitro* cultures. The first culture was grown in ARS medium supplemented with antibiotics at the above concentrations (control group). The second was grown in a saturated solution of unlabelled IVM (11.44 *μ*M) in ARS supplemented with antibiotics (study group, IVM). The start group (S) consisted of washed living worms before the foundation of the cultures. Single individuals were cultured in sterile 50 mL culture flasks at 37°C under 5% CO_2_ in air. During the culturing the motility of the parasites was observed. After 4, 8, and 20 hours samples of muscle from 5 worms were taken equally from both cultures under sterile conditions. Then, samples for enzyme assays and molecular analysis were frozen in liquid nitrogen and stored at −70°C.

### 2.3. Enzyme Assays

Samples of prepared muscle tissue were homogenized in an Omni TH-02 (5,000–35,000 revolutions per min; Omni International, USA) homogenizer in a 1 : 4  (w/v) ratio in 0.9% NaCl. The homogenates were centrifuged at 1500** **g for 15 min at 4°C using an Eppendorf Centrifuge 5804 R (USA). The supernatant fraction was a crude extract used for enzyme assays. The activity of TPS and TPP was determined using modified methods described by Giaever et al. [23] and Kaasen et al. [24], respectively. The reaction mixture for TPS activity contained 0.2 mL of 0.1 M acid-ammonia buffer (pH 4.2), 0.1 mL of 10 mM MgCl_2_, 50 *μ*L of 2 mM UDPG, 50 *μ*L of 2 mM G6P, and 0.1 mL of the crude extract of enzyme. After mixing, appropriate control samples were incubated at 100°C for 5 min; tested samples were incubated at 37°C for 30 min and after that at 100°C for 5 min to complete the reaction. In a second step, the resulting T6P was decomposed by the addition of 0.1 mL of the alkaline phosphatase solution (1 U) in 0.1 M phosphoric buffer (pH 8.0) at 37°C for 30 min. The reaction mixture for TPP activity contained 0.2 mL of 0.1 M phosphoric buffer (pH 7.0), 0.1 mL of 10 mM MgCl_2_, 0.1 mL of 2 mM T6P, and 0.1 mL of the crude extract of enzyme. After mixing, appropriate control samples were incubated at 100°C for 5 min; tested samples were incubated at 37°C for 60 min and after that at 100°C for 5 min to complete the reaction. The end product of the activities of both the enzymes, trehalose, was assayed by high-performance liquid chromatography (HPLC) using a method described by Dmitryjuk et al. [13]. The activities of TPS and TPP were expressed in units (U) per mg protein measured using the Bradford [25] method with BSA as a standard. One unit defines the quantity of trehalose (nmol) formed during 1 min at 37°C.

### 2.4. Real-Time Quantitative Polymerase Chain Reaction

Total RNA extraction was performed using a Total RNA Kit according to the manufacturer's protocol (A&A Biotechnology). The RNA was spectrophotometrically quantified (A260 nm), and its quality was determined by gel electrophoresis on 1% agarose containing 0.01% ethidium bromide. A TranScriba Kit was used for first-strand cDNA synthesis. First, 1 *μ*g of total RNA was preincubated with 1 *μ*L of 10 mM appropriate reverse primer (Table 1) in a total volume of 10 *μ*L (in the nuclease-free water) in an Applied Biosystems Veriti 96-well Thermal Cycler (Life Technologies, USA) at 65°C for 5 min to denature the RNA template secondary structures and then cooled on ice for 5 min. After that, 4 *μ*L of 5x reaction buffer, 2 *μ*L of 10 mM mix of triphosphate deoxyribonucleotides (dNTPs), and 4 *μ*L of TranScriba reverse transcriptase (20 U/*μ*L) were added to the mixture and incubated at 41°C for 60 min and for the next 5 min at 70°C. Real-time reverse transcriptase qPCR (qRT-PCR) was performed using SYBR Green B PCR-MIX Taq. The reaction mixture consisted of 2 *μ*L cDNA, 5 *μ*L SYBR Green PCR-MIX, 1 *μ*L 10 mM of both appropriate forward and reverse primers (Table 1), and 0.15 *μ*L ROX (5-carboxy-X-rhodamine) reference dye, and the volume was raised to 10 *μ*L with nuclease-free water. Mean values and standard deviations were used for the analysis of relative transcript levels for each time point using the 2^−ΔΔC_T_^ method [26]. The data were presented as the fold change in gene expression normalized to an endogenous reference gene* gapdh* (glyceraldehyde-3-phosphate dehydrogenase; housekeeping gene; Table 1) and relative to the untreated control (relative quantification (RQ) = 1; start group or control group). Transcript levels were shown by AB analysis software (7500 v2.0). All samples were tested in triplicate on Applied Biosystems 7500 Fast Real-Time PCR Systems (Life Technologies, USA). Melting curves were constructed after amplification.

### 2.5. Statistical Analysis

Data were expressed as means ± standard deviation by one-way ANOVA using Statistica 10 (StatSoft Inc., Tulsa, Oklahoma, USA). Tukey's honestly significant different (HSD) test was used to assess differences between means. Pearson's correlation test was used for determination of correlation between TPS or TPP relative activity and their mRNA expression over time. The *P* value less than 0.05 (*P* < 0.05) was considered statistically significant. All the experiments were performed in triplicate.

## 3. Results

### 3.1. Observation of the Mobility of Worms during* In Vitro* Cultivation

Worms in the control group were highly mobile to the end of the* in vitro* cultivation. However,* A. suum* in the IVM medium displayed a gradual decrease in motility. After 4 hours IVM roundworms showed negligible mobility. Paralysis of the worms increased further after 8 hours of the culture. The* in vitro* culture was terminated after 20 hours, when the roundworms from the IVM-group did not show any movement.

### 3.2. Activity of Enzymes in the Trehalose Synthesis Pathway

**Figure 1:**
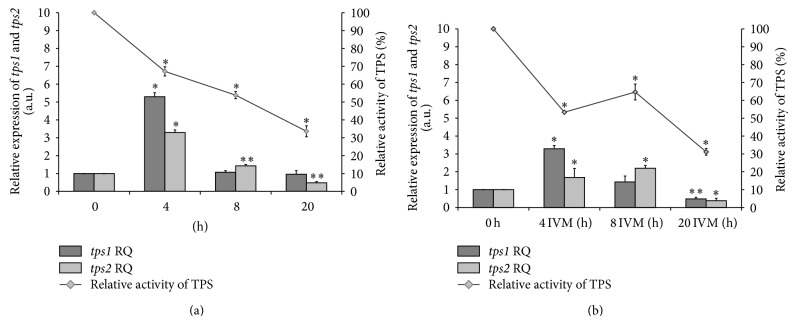
Relative activity of trehalose-6-phosphate synthase (TPS) and its mRNA (*tps1*,* tps2* genes) expression normalized to the endogenous reference* gapdh* (glyceraldehyde-3-phosphate dehydrogenase) gene and relative to the expression at time zero (start group). IVM: ivermectin; RQ: relative quantification; (a) control group; (b) IVM-group; ^*^significant change at *P* < 0.01 and ^**^significant change at *P* < 0.05 with respect to the start group.

**Figure 2:**
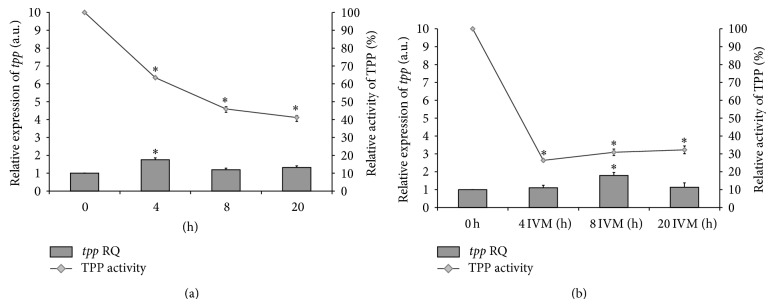
Relative activity of trehalose-6-phosphate phosphatase (TPP) and its mRNA (*tpp* gene) expression normalized to the endogenous reference* gapdh* (glyceraldehyde-3-phosphate dehydrogenase) gene and relative to the expression at time zero (start group). IVM: ivermectin; RQ: relative quantification; (a) control group; (b) IVM-group; ^*^significant change at *P* < 0.01 with respect to the start group.

TPS and TPP activities were observed during 20 hours of* in vitro* cultivation of adult female* A. suum* (Table 2). In the case of TPS, a gradual decrease in the activity was observed in the control group. Differences between means were statistically significant here. After 20 hours of incubation, the enzyme showed activity equal to 33.64 ± 3.07% of the starting (Table 2; Figure 1(a)). In the group supplemented with IVM a significant decrease in the activity of TPS was initially observed (after 4 hours to 54.12 ± 1.74%). After 8 hours of incubation in IVM-group was observed a slight increase of TPS activity relative to 4-hours culture (up to 64.64 ± 4.43%) in contrast to the control group where a further decrease in enzyme activity was noted. After 20 hours of culture, again there was a greater decrease to a value slightly lower than that of the control group (31.13 ± 1.85%). Also, there were statistically significant differences compared with the means of the start (S) group in all groups (Figure 1(b)). TPP activity in the IVM-group was lower than that in the control group, after 4, 8, and 20 hours of incubation of the worms. In addition, the activity of TPP decreased during incubation in the control group and these changes were statistically significant (Figure 2(a)) but slightly increased in the IVM-group after 8 and 20 hours (Table 2; Figure 2(b)).

### 3.3. Real-Time Quantitative Polymerase Chain Reaction

#### 3.3.1. Expression of Trehalose-6-phosphate Synthase and Phosphatase mRNA Relative to Expression at Time Zero (Start Group)

TPS mRNA was expressed in all groups. However, differences in the expression of* tps1* and* tps2 *genes were noted during the* in vitro* culturing of* A. suum* (Figures 1(a) and 1(b)). Initially,* tps1* expression in both groups increased several times relative to the expression at time zero and this was a statistically significant increase. After 8 hours, expression of the gene slightly increased and then decreased again after 20 hours in IVM-group relative to the control group (Figures 1(a) and 1(b)). Between* tps1 *gene expression and TPS activity was observed a fairly strong correlation in the control group (*r* = 0.8009) and a moderate correlation (*r* = 0.5074) in the case of IVM-group with respect to the start (Table 2).

Expression of the* tps2* gene increased in both groups, but the increase of this gene expression was about half lower in the group cultured in medium supplemented with IVM than in the control group. In both cases statistically significant differences were noted. After 8 hours of incubation in the IVM medium,* tps2* expression level was higher than at the start and control (RQ = 2.2 ± 0.16; Figures 1(a) and 1(b)). Between* tps2 *gene expression and TPS activity was observed a weak correlation in the control group (*r* = 0.3576) and a very strong correlation (*r* = 0.9344) in the case of IVM-group (Table 2).

The expression level of the gene encoding TPP also fluctuated during worm cultivation (Figures 2(a) and 2(b)). After 4, 8, and 20 hours of incubation,* tpp* expression level was a little higher relative to the start. The highest level of gene expression was achieved after eight-hour incubation in IVM-group (RQ = 1.79 ± 0.16). At the end of the culture, this gene expression increased in the control group; however, in the group treated with IVM* tpp* expression dropped to the level of start expression (RQ = 1.12 ± 0.25; Figures 2(a) and 2(b)). A fairly strong correlation (*r* = 0.8680) in the control group and a weak correlation (*r* = 0.3645) in the case of IVM-group between* tpp *gene expression and TPP activity were observed (Table 2).

#### 3.3.2. Expression of Trehalose-6-phosphate Synthase and Phosphatase mRNA Relative to the Control Groups

**Figure 3:**
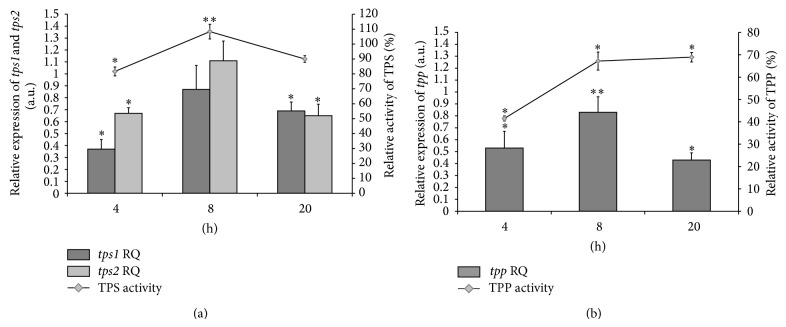
Relative activity of trehalose-6-phosphate synthase (TPS) and phosphatase (TPP) and their mRNA expression (*tps1*,* tps2*, and* tpp* genes) normalized to the endogenous reference* gapdh* (glyceraldehyde-3-phosphate dehydrogenase) gene and relative to the control groups. IVM: ivermectin; RQ: relative quantification; (a) relative activity of TPS and* tps1*,* tps2* expression; (b) relative activity of TPP and* tpp* expression; ^*^significant change at *P* < 0.01 and ^**^significant change at *P* < 0.05 with respect to the control group.

A subsequent real-time quantitative PCR analysis tested the expression of trehalose synthesis genes relative to the control groups. The* tps1* expression was always at a lower level relative to the control group. Initially,* tps1* gene expression was the lowest among the tested genes (RQ = 0.37 ± 0.08). After 8 hours expression increased to RQ = 0.86 ± 0.2, but after 20 hours it decreased to RQ = 0.69 ± 0.07 (Table 2). Differences between means for the IVM-group after 4 and 20 hours of culture were statistically significant with respect to the control (Figure 3(a)). Between* tps1 *gene expression and TPS activity was observed a fairly strong correlation (*r* = 0.7493; Table 2).

Expression of the* tps2* gene after 4 and 20 hours was lower than that of the control group (RQ = 0.67 ± 0.047 and RQ = 0.65 ± 0.096, resp.). After 8-hour incubation expression of this gene was a little higher than in the control but it was not a statistically significant change relative to the control (RQ = 1.11 ± 0.16; Table 2; Figure 3(a)). As with* tps1*, between* tps2 *gene expression and TPS activity was observed a fairly strong correlation (*r* = 0.8108; Table 2).

The level of TPP mRNA expression was lower relative to the control group during the cultivation. The highest expression of* tpp* was also observed after 8 hours of the culture (RQ = 0.83 ± 0.13; Table 2; Figure 3(b)). The weak Pearson's correlation was here between* tpp* gene expression and TPP activity (*r* = 0.3257; Table 2).

## 4. Discussion

Trehalose metabolism may provide a new target for combating parasitic nematodes in mammals [27, 28]. This is particularly true for the destruction of the T6P pathway, which is likely to be toxic for nematodes. As mentioned in the Introduction, Kormish and McGhee [19] discovered* gob-1* lethality in* C. elegans*, which is not due to low trehalose levels but rather to the buildup of the intermediate T6P. In addition, mutations in* tps1* and* tps2* almost completely suppress the* gob-1* loss-of-function phenotype. The results of these studies suggest that TPP could be a good target for new anthelmintic drugs. Promising results were obtained by Kushwaha et al. [21], who investigated the silencing of* B. malayi* TPP* in vitro*. Silencing of the* tpp* gene had the effect of reducing fertility in* B. malayi* males and females, resulted in violations of embryogenesis in the interior of females, was lethal to L_3_ larvae, and led to fatal effects during the development of the parasite in the host. In the present research, we decided to investigate if there would be a similar effect to the above on the trehalose synthesis pathway with the use of the antiparasitic drug IVM in* in vitro* cultures of mature female* A. suum*.

IVM is a widely used antiparasitic agent. It was used in* in vitro* cultures of adult male cattle parasite* Onchocerca ochengi* [29]. Previous research has used, like us, a saturated solution of unlabelled IVM (11.44 *μ*M) for the cultivation of adult parasites. Although it was not a lethal dose for* O. ochengi*, it is the drug concentration administrated to pigs in feed and is sufficient to kill nonfilarial nematodes. In* O. ochengi* uptake of IVM was high by 3 hours of exposure, and uptake continued for up to 12 hours. This process occurred predominantly by the transcuticular route. In our study, after only 4 hours of IVM exposure, we noticed a significant paralysis and loss of mobility in the adult female* A. suum*. In the subsequent hours of incubation, worm shock deepened. After 20 hours, the roundworms were straight and showed no movement, while in the control worms we observed complete motility.

In the available literature there are several reports regarding IVM efficacy against larval and adult* A. suum* and* A. lumbricoides in vivo* [10, 11, 30, 31]. It is likely that IVM does not penetrate the shell of parasite eggs, because the larvae that develop in the eggs from IVM-treated groups appear fully infective for mice [9]. The impact of IVM on the trehalose metabolism of parasitic organisms seems particularly interesting, because this disaccharide acts as a key survival strategy of nematodes during exposure to various environmental stressors [15]. In addition, these studies are part of the current trend of the antiparasitic drugs research, which mostly revolve around pathways of trehalose synthesis. For example, Farelli et al. [32] recent results showed that structure of the trehalose-6-phosphate phosphatase from* B. malayi* reveals key design principles for anthelmintic drugs.

It is known that the ivermectin was located in the outer monolayer of the* A. suum* muscle membrane and nerve cord [7]. In our studies, we showed that IVM affects the profile of enzyme activity of the trehalose synthesis pathway and the expression of the mRNA of these enzymes in the muscle of* A. suum*. However, it is worth noting that the drug does not block the metabolic pathway. In the control groups, we observed a gradual decrease in the activity of both TPS and TPP in the muscle of the parasites. This may be because the culture medium does not contain nutritional components. TPS activity increased after 8 hours of incubation in the IVM-group in relation to the 4-hour cultures of IVM-group and control. It is possible that stress induced by the administration of IVM was stronger than starvation and affects T6P synthesis from accumulated reserve components such as glycogen. It is also clear that an efficient excretory system would detoxify the parasite and make it less susceptible to the therapeutic action of drugs [7]. The fact that* tps2* expression was two times higher relative to the start and slightly rose relative to the control, at relatively lower expression of the* tps1* gene, leads to the conclusion that the* tps2* gene here is responsible for the increased activity of TPS in worms being IVM-treated here at this time.

In both groups there was a positive correlation between gene expression and the specific enzymes activity relative to the start. The highest level of correlation between the activity of a specific enzyme and the gene expression was observed for the* tps2* gene at IVM-group, while, in the control group, this correlation was weak. It was observed a high correlation of the* tps1* gene expression with the TPS activity in the control group and moderate in IVM-group with respect to the start. In the case of* tpp*, the correlation between gene expression and the TPP activity was strong in the control group and weak in the IVM-group.

Relative to the appropriate control groups we noted the highest correlation coefficient between the activity of a specific enzyme with* tps2* gene expression. Also in the case of* tps1* there was fairly strong correlation. Between the activity of TPP and the* tpp* expression relative to control was observed a weak correlation, as in the case of analysis with respect to the starting. To summarize, we can say that T6P synthase activity in the IVM-group is positively correlated mainly with the* tps2* gene expression. In the case of T6P phosphatase the same relationship was not observed.

Expression profiles of mRNA were different for each of the three examined genes. There was a decrease in* tps1* and* tps2* gene expression after 20 hours in the IVM medium, relative to the expression at time zero. However, the level of* tpp* expression at this time was very similar to the start. Relative to the control, the expression level was lower for all three genes at the end of the culture; however, expression of* tps2 *was a little higher after 8 hours in the IVM-group. Results of this study could suggest that this concentration of IVM acts mainly by lowering the expression of the* tps1* and* tpp* genes. It is worth noting that in the 8-hour incubation in IVM medium an increase in expression of all three genes in relation to measurements occurred at 4 hours of incubation. In the case of* tpp* gene it was an increase above the control value. In addition, it is positively correlated with increased activity of trehalose synthesis enzymes. The 8-hour incubation seems to be a key moment of the impact of IVM to stimulate the metabolism of trehalose in adult worms. Further culturing worms in the drug solution caused a rapid decreasing of studied parameters. Given that the decrease in activity of a satisfactory effect of the enzymes and their mRNA expression was obtained after 20 h of incubation, when the worms were completely nonmotile, the effect of the drug on the trehalose metabolism of adult worms present in the intestine of the host* in vivo* appears to be negligible, because IVM enters the blood as early as 4 hours after oral administration [2]. It seems that IVM may more negatively affect the trehalose synthesis of* A. suum* larvae in host body.

## 5. Conclusions

Based on the results of our research it can be concluded that the search for new drugs that block the trehalose synthesis path in* A. suum* is justified to enhance the action of ivermectin and maybe another drugs. Addition of trehalose synthesis pathway inhibitors can contribute to more effective control of the parasites in the future. The study also confirms that trehalose metabolism is activated to protect against lethal effects of the drug in the 8 hours of cultivating and this process is regulated at the molecular level. In addition, it correlates positively with the activity of specific enzymes. We can also say that trehalose synthesis pathway is activated in stress conditions, more during treatment with the use of antiparasitic drug than starvation.

Although the lethal dose of IVM reduces mRNA expression of trehalose synthesis enzymes by roundworms cultivated* in vitro*, it does not block the path completely and the time necessary to obtain a satisfactory result is too long. Because of this and the progressive drug resistance of parasites, it seems reasonable to study trehalose synthesis pathway gene silencing in the future.

## Figures and Tables

**Table 1:** Primer sequences.

Enzyme	Gene	Primer name	Sequence (5′ → 3′)	GenBank accession number of target sequence
Trehalose-6-phosphate synthase (TPS)	*tps1 *	Forward	CTCGTCGTTGTGCACAGTTT	JF412033.2
		Reverse	GCTGTTCCAGCAGTTCTTCC	
	*tps2 *	Forward	CCACATGCGGGTCTTATTCT	JF412034.2
		Reverse	ACTCTCGGTGGCTGCACTAT	

Trehalose-6-phosphate phosphatase (TPP)	*tpp *	Forward	CATGGCACTCTTTGTTGGTG	JI169681.1
		Reverse	AGCGTTATTCAGTGCCTCGT	

Glyceraldehyde-3-phosphate dehydrogenase (GAPDH)	*gapdh *	Forward	CGGTTGTATCGACGGACTTT	AB058666.1
		Reverse	TGAGGCTTTGACGTTCAGTG	

**Table 2:** Activity of trehalose-6-phosphate synthase (TPS) and phosphatase (TPP) and their mRNA expression (*tps1*, *tps2, *and *tpp* genes) during *in vitro* cultivation of adult female *Ascaris suum* in control (C-group) and ivermectin-supplemented medium (IVM-group).

Group of worms	Activity of TPS [nM/mg]	Activity of TPS relative to the start [%]	*tps1* RQ to the start	*tps2* RQ to the start	Activity of TPS relative to the control [%]	*tps1* RQ to the control	*tps2* RQ to the control
S-group	420.20 ± 4.0	100	1	1	—	—	—
4 h C-group	295.41 ± 11.3	67.13 ± 2.56	5.3 ± 0.21	1.68 ± 0.15	100	1	1
8 h C-group	236.97 ± 8.9	53.85 ± 2.03	1.06 ± 0.09	1.55 ± 0.06	100	1	1
20 h C-group	148.04 ± 13.5	33.64 ± 3.07	0.96 ± 0.19	1.59 ± 0.06	100	1	1
Correlation coefficient			**r** = 0.8009^*^	**r** = 0.3576^*^			
4 h IVM-group	238.26 ± 7.65	54.12 ± 1.74	3.29 ± 0.17	1.67 ± 0.49	81.63 ± 3.02	0.37 ± 0.08	0.67 ± 0.04
8 h IVM-group	256.73 ± 11.7	64.64 ± 4.4	1.42 ± 0.33	2.2 ± 0.16	108.32 ± 4.94	0.87 ± 0.20	1.11 ± 0.16
20 h IVM-group	133.23 ± 3.3	31.13 ± 1.85	0.48 ± 0.09	0.37 ± 0.12	89.99 ± 2.22	0.69 ± 0.07	0.65 ± 0.09
Correlation coefficient			**r** = 0.5074^*^	**r** = 0.9344^*^		**r** = 0.7493^**^	**r** = 0.8108^**^

Group of worms	Activity of TPP [nM/mg]	Activity of TPP relative to the start [%]	*tpp* RQ to the start	Activity of TPP relative to the control [%]	*tpp* RQ to the control		

S-group	746.61 ± 85.2	100	1	—	—		
4 h C-group	473.95 ± 6.3	63.48 ± 0.84	1.75 ± 0.1	100	1		
8 h C-group	343.24 ± 9.9	45.97 ± 1.33	1.19 ± 0.09	100	1		
20 h C-group	310.47 ± 5.1	41.13 ± 1.15	1.32 ± 0.08	100	1		
Correlation coefficient			**r** = 0.8680^*^				
4 h IVM-group	196.95 ± 5.5	26.35 ± 0.74	1.1 ± 0.14	41.51 ± 1.17	0.53 ± 0.14		
8 h IVM-group	230.58 ± 13.8	30.88 ± 1.85	1.79 ± 0.16	67.18 ± 4.03	0.83 ± 0.13		
20 h IVM-group	213.95 ± 6.58	32.26 ± 2.18	1.12 ± 0.25	68.9 ± 2.12	0.43 ± 0.06		
Correlation coefficient			**r** = 0.3645^*^		**r** = 0.3257^**^		

^*^Pearson's correlation coefficient between gene expression and appropriate enzyme activity changes over time with respect to the start group (S); ∗∗Pearson's correlation coefficient between gene expression and appropriate enzyme activity changes over time with respect to the C-group.
